# Supplementary material for: Evidence Gaps and Lessons in the Early Detection of Atrial Fibrillation: A Prospective Study in a Primary Care Setting (PREFATE Study)
Source: Biomedicines. 2025 Jan 7;13(1):119. doi: 10.3390/biomedicines13010119 (PMC11759169; doi:10.3390/biomedicines13010119)
Supplement: Supplementary file 1 [file biomedicines-13-00119-s001.zip › biomedicines-3398781-supplementary.pdf]

Table S1. MVP ECG risk score and probability of AF basis on the punctuation [20].

P-WAVE VARIABLE	VALUE	SCORE
**Morphology in inferior leads**	Nonbiphasic < 120ms	0
	Nonbiphasic ≥ 120ms	1
	Biphasic	2
**Voltage in lead I**	>0,2 mVv	0
	O,1-0,2 mV	1
	< 0,1mV	2
**Duration**	< 120ms	0
	120-140 ms	1
	>140 ms	2

Probability of AF: . low: 0-1 . intermediate: 3-4 (Odds ratio 2,1) high: 5-6 (Odds ratio 2,4)

Table S2. Normal vàlues and ranges of LA strain components [21].

LA STRAIN COMPONENT	Nº OF STUDIES	MEAN	95% CI	COCHRANE Q
Reservoir	40	39,4	38,0–40,8	1,653 (p < 0,001)
Conduit	14	23,0	20,7–25,2	420 (p < 0,001)
Contractile	18	17,4	16,0–19,0	631 (p < 0,001)
